# Preparation and Characterization of *Aronia melanocarpa*/Gellan Gum/Pea Protein/Chitosan Bilayer Films

**DOI:** 10.3390/foods11182835

**Published:** 2022-09-13

**Authors:** Xuanhong Chen, Wei Ji, Xijun Nan, Honglei Wang, Jiayi Li, Leichao Dong, Guihua Sheng, Quancheng Zhou

**Affiliations:** 1 Department of Food Science, School of Agricultural Engineering and Food Science, Shandong University of Technology, Zibo 255049, China; 2 Key Laboratory of Shandong Provincial Universities for Technologies Agricultural Products, Zibo 255049, China

**Keywords:** *Aronia melanocarpa* (AM), gellan gum (GG), food freshness indicator, pH-sensitive bilayer films, film mechanical characterizations, food weight loss

## Abstract

In this study, pH-sensitive bilayer hydrogel films with different AM contents (0.00%, 0.50%, 1.00%, 1.50%, 2.00% and 2.50%) were constructed. The films took AM/GG hydrogel as the inner layer structure and a pea protein (PP)/chitosan (CS) composite system as the outer structure. Film formation and the effect of AM were clarified through the detection and analysis of mechanical properties, microstructure, pH sensitivity and fresh-keeping ability. Results showed that AM exhibited good compatibility with each substance in the composite film, which were evenly dispersed in the system. The addition of AM significantly improved the water content, tensile strength, elongation at break, puncture resistance, oil resistance and water resistance of the composite films. The antioxidant activity, pH sensitivity and fresh-keeping effect of the composite film on fresh pork were remarkably enhanced. Moreover, it was found that the composite film containing AM effectively inhibited the production of total volatile base nitrogen (TVN) in fresh pork and significantly reduced the weight loss of fresh pork due to water loss during storage. Therefore, the functional properties revealed that AM was more positive to the comprehensive performance of films, and the AM-GG/PP-CS bilayer film containing AM exhibited strong potential for use in food preservation and packaging as a food freshness indicator to test food quality changes in storage.

## 1. Introduction

Recently, consumers have put forward higher requirements for food packaging materials due to the increased demand for safe, fresh and high-quality food [1]. Food packaging should protect food, facilitate transportation and be safer and healthier. Furthermore, the ability to preserve food quality is extremely important. Thus, food freshness intelligent indication packaging film is a research hotspot at present [2].

*Aronia melanocarpa* (AM), a natural pigment, is rich in antioxidant components (anthocyanins, flavonoids, total phenols) [3,4]. Studies have shown that AM plays a role in food preservation, effectively improving the microbial composition of pork products during storage [5]. In addition, AM can prepare PU/AM nanofibers with excellent performance by forming hydrogen bonds with polyurethane (PU), which is attributed to its improved physical and chemical properties [6]. Therefore, AM has potential application value in the construction of food preservation packaging film, but there are few research reports on it.

Gellan gum (GG), as an exopolysaccharide, possesses the characteristics of nontoxicity and easy degradation. It can form a gel with excellent inclusion and film formation properties [7]. A previous study showed that packaging film for the intelligent indication of food freshness can be prepared by combining GG with artemisinin gum and blueberry anthocyanin [8]. However, the practical application value of thin film material is reduced, which is attributed to the disadvantage that thin film material is susceptible to the influence of the external environment pH and has poor mechanical properties.

Therefore, the objective of this study was to develop an intelligent food freshness indicator packaging film with good mechanical properties and sensitivity to pH discoloration. As a preservative and modifier of the film-forming material, AM was taken to construct an AM-GG composite film. Furthermore, pea protein (PP)/chitosan (CS) composite film as the outer layer of AM-GG was constructed to protect the AM-GG performance from the influence of the external environment. The mechanical properties, microstructure, pH sensitivity and fresh-keeping properties of the films were analyzed by one-way analysis of variance (ANOVA, *p* ≤ 0.05) to clarify the structural law of the films and reveal the effect of AM on the films.

## 2. Materials and Methods

### 2.1. Materials

AM fruits were collected from the Zibo Forestry Protection and Development Center (Zibo, China). Pea protein was purchased from Shuangta Food Co., Ltd. (Yantai, China). Gellan gum (GG) was food grade (FG) and procured from Kelin Biological Technology Co., Ltd. (Shanghai, China). Hydrochloric acid (analytical reagent (AR)), sodium hydroxide (AR), potassium bromide (AR), sodium chloride (AR), chitosan (FG), calcium chloride (FG) and glycerin (FG) were procured from Shanghai Aichun Biological Technology Co., Ltd. (Shanghai, China). Glacial acetic acid was purchased from Cuhk Food Ingredients Co., Ltd. (Zhejiang, China). Distilled water was used as a solvent for the preparation of the film solution. 

### 2.2. Film Preparation

#### 2.2.1. Preparation of AM-GG Composite Film Solution

Calcium chloride of 111.00 mg and glycerol of 2.50 mL were added into 100.00 mL GG solution (1.00% (*w*/*v*)) [9,10], and then 0.00, 0.50, 1.00, 1.50, 2.00 and 2.50 mL AM juice were added. The AM-GG composite film solution was prepared by ultrasonic defoaming for 10 min after stirring.

#### 2.2.2. Preparation of PP-CS Composite Film Solution

PP solution preparation [11]: PP solution with a mass fraction of 2.00% (*w*/*v*) was prepared by ultrasonic water bath stirring at 50 °C. CS solution configuration: 4.00× *g* CS powder was added to 200.00 mL 1.00% (*v*/*v*) edible acetic acid solution and stirred in the water bath at 50 °C to prepare 2.00% (*w*/*v*) CS solution. PP-CS composite film solution was prepared by stirring CS and PP solution with a mixing ratio of 2:1 and 1.50 mL glycerol for 3 h.

#### 2.2.3. Preparation of AM-GG/PP-CS Bilayer Films

A 50.00 mL volume of the AM-GG solution was slowly poured onto a square petri dish (13 cm × 13 cm) and dried in an incubator at 25 °C until a firm but still sticky surface was obtained. Then, 50.00 mL of the PP-CS solution was naturally cast onto the AM-GG layer. After drying in an incubator at 30 °C for 48 h, the film was removed from the square petri dish and used for subsequent experiments.

### 2.3. Fourier-Transform Infrared Spectroscopy (FT-IR)

FT-IR of the film was tested according to Chen et al. [12]. with slight modifications. FT-IR spectra were recorded on a Nicolet 5700 infrared spectrophotometer (Thermoelectric Nicoli Instrument Company, Atkinson, NH, USA) with 32 scans at a resolution of 2 cm^−1^ in the wavenumber range of 4000–400 cm^−1^.

### 2.4. Microstructure of Films

According to Chang et al. [13], the samples were first immersed in liquid nitrogen and fractured, then sputter-coated with gold. Then, the morphology and the cross-section microstructure of the film were observed by a scanning electron microscope (SEM, Quanta 250, FEI Company, Hillsboro, OH, USA) at an accelerating voltage of 5 kV and recorded at 2000 magnifications.

### 2.5. Physical Properties of Films

#### 2.5.1. Film Thickness

According to Wahidin et al. [14], the thickness of the film was measured by using a flat-head thickness gauge (Shenzhen Yuanhengtong Technology Co., Ltd., Shenzhen, China). Five test points were selected around and in the center of each sample, and $\overset{-}{d}$ as the film thickness was calculated according to the average of 5 random measurement points.

#### 2.5.2. Moisture Content (MC)

The gravimetric technique was used to determine the MC of the films [15]. MC was calculated using Equation (1).

(1) $$MC/\%\;=\;\frac{m_{1}-m_{2}}{m_{1}}\;\times \;100\%$$

where m_1_ and m_2_ are the weight of samples before and after drying, respectively.

#### 2.5.3. Mechanical Properties

The mechanical properties of the films, such as tensile strength (TS) and elongation at break (EB, %), were measured based on the GB/T 1040.3–2006 [16] standard by using the TMS 2000 Texture Analyzer (Fts International, Inc., Fort Worth, TX, USA). For this, the film samples were cut into rectangular pieces of about 4.00 cm × 2.00 cm, and food-grade PE film was the control. Initial grip distance was set to 20 mm with a crosshead speed of 1.00 mm/s. TS (N/mm) and EB (%/mm) were calculated by Equations (2) and (3), respectively. The average value was calculated from at least three measurements for each film.

(2) $$TS\;=\;\frac{F}{\overset{-}{d}}$$

(3) $$EB\;=\;\frac{\left(L_{1}\;-\;L_{0}\right)}{\left(L_{0}\;\times \;\overset{-}{d}\right)}\;\times \;100\%$$

where F is the maximum force at break (g), and $\overset{-}{d}$ is the film thickness (mm). L_0_ and L_1_ are the lengths of the film piece before and after stretching (mm), respectively.

#### 2.5.4. Puncture Resistance

Puncture resistance of the film was tested according to GB/T 37841-2019 [17]. Each film sample was cut into a rectangular piece about 6.00 cm × 4.00 cm and then mounted on a TMS 2000 Texture Analyzer (Fts International, Inc., USA). The puncture speed was 10.00 mm/min. Puncture resistance (N/mm) was calculated by Equation (4). The average value was calculated from at least three random measurements for each film.

(4) $$\mathrm{Puncture}\;\mathrm{resistance}\;=\;\frac{F_{1}}{\overset{-}{d}}$$

where F_1_ is the maximum force at puncture (g), and d is the film thickness (mm). 

### 2.6. Water Vapor Permeability (WVP)

The WVP of the film was determined based on the method of Zou et al. [1], with slight modifications. Each film was cut and sealed to the opening of a 50 mL beaker containing 20.00 mL distilled water. Then, the beaker was placed in a desiccator with a temperature of 22 °C and containing completely dried silica gel. The weight change of the beaker was recorded every hour until it reached a constant weight. The WVP (g·mm/(m^2^·s·Pa)) of the films was determined by Equation (5). Three replications of each film were tested for the WVP.

(5) $$WVP\;=\;\frac{(m_{0}\;-\;m_{1})\;\times \;x}{S\;\times \;\Delta P\;\times \;t}$$

where t is the elapsed time (s), m_0_ is the initial weight (g) and m_1_ is the weight after t hours (g), S is the area of the tested film (m^2^), x is the film thickness (mm) and ΔP is the partial vapor pressure difference between the dry atmosphere and pure water.

### 2.7. Oil Permeability (OP)

The OP of the film was determined based on the method of Yan et al. [18], with slight modifications. Salad oil of 3.00 mL was added to a test tube and then sealed with the specimen films. The tube was turned upside down on the filter paper and placed into a desiccator for seven days. The weight change of the beaker was recorded every day. The OP (g·mm/(m^2^·d)) of the films was determined by Equation (6). Three replications of each film were tested for the OP.

(6) $$OP=\frac{\Delta W\times D}{S\times T}$$

where ΔW is the weight change of filter paper (g), T is the elapsed time (d), S is the area of contact between the film and the oil (m^2^) and D is the film thickness (mm).

### 2.8. Color Change of Film at Different pH Environments

The color change of the film was determined based on the method of Zou et al. [1]. The prepared bilayer film was cut into a 4.00 cm × 4.00 cm size. The WSD-3C whiteness meter (Beijing Kangguang Optical Instrument Co., Ltd, Beijing, China). was applied to measure the brightness (L*), red-green (a*) and yellow-blue (b*) of the film. The film was added to the pH standard solution with pH values of 2, 4, 7, 10 and 12. After standing for 30 s, the L_0_*, a_0_* and b_0_* values of the film were measured again. Whiteness (ΔE) was calculated using the following formula:

(7) $$\Delta E=\sqrt{{(\Delta L*}^{2}+{(\Delta a*}^{2}+{(\Delta b*}^{2})}$$

where ΔL* = L* − L_0_*, Δa* = a* − a_0_* and Δb* = b* − b_0_*.

### 2.9. Fresh-Keeping Effect

In a sterile room, fresh minced pork of 40.00 g was placed on a sterile culture dish, which was covered with AM-GG/PP-CS bilayer edible composite film with different AM contents (fresh minced pork was covered by AM-GG film, and PP-CS film was exposed to air). Then, the culture dish was placed into a 4 °C refrigerator for storage. The content of TVN in fresh pork was detected by a test kit at 0, 1, 2, 3, 4, 5, 6 and 7 d. Furthermore, the weight of fresh pork on different days was measured and recorded. The experimental group without any fresh-keeping treatment was used as the blank control, and the experimental group with food fresh-keeping film was used as the positive control. The other operation steps were the same. The experiment was repeated three times, and the results were averaged. The weight loss ratio of fresh pork was calculated according to the following formula.

(8) $$W=\frac{\left(M_{0}-M_{1}\right)}{M_{0}}\times 100\%$$

where W is the weight loss ratio of fresh pork, M_1_ is the remaining weight of fresh pork on different days and M_0_ is the initial weight of fresh pork.

### 2.10. Experimental Design and Statistical Analysis

All tests were conducted in a completely randomized design in independent triplicates to confirm the reproducibility of the results. The report of the data was given as mean ± SD [19]. A one-way ANOVA was performed to assess specific data. The differences among the experimental groups were analyzed through a one-way analysis of variance (ANOVA, *p* ≤ 0.05), followed by Dunnett’s test. 

## 3. Results and Discussion

### 3.1. FT-IR Spectra

**Figure 1:**
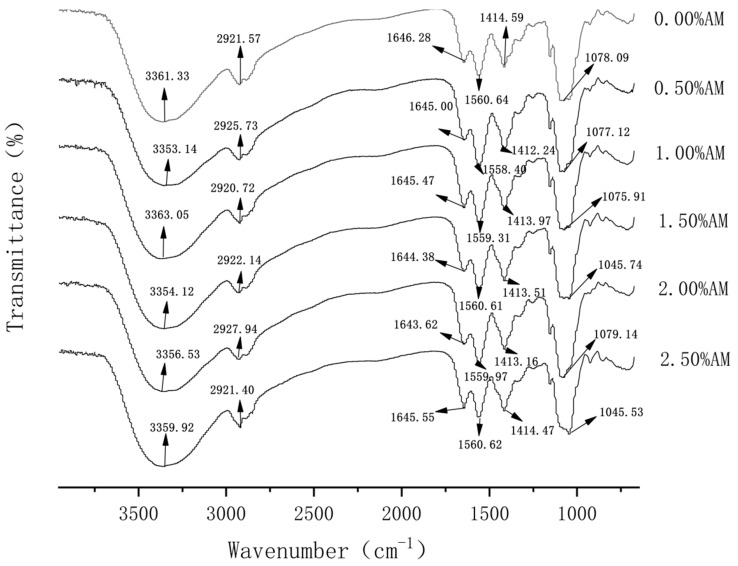
FT-IR of AM-GG/PP-CS bilayer films.

FT-IR spectra play an important role in the analysis of molecular structure. The width, intensity and position of the absorption peak can reflect the conformational change of the substance at the molecular level. When different polymers are compatible, intermolecular interactions often occur, which are manifested as changes in spectral band information. Therefore, FT-IR analysis is beneficial to elaborate on the degree of intermolecular compatibility of polymers [20]. As shown in Figure 1, 0.00% AM films had obvious absorption peaks at 3361.33 cm^−1^ and 1646.28 cm^−1^, which were generated by the stretching vibration of -OH and -CO groups, respectively [21]. After the addition of AM, the absorption peaks at 3361.33 cm^−1^ and 1646.28 cm^−1^ moved to the lower wavenumber direction, indicating that AM and GG are held together by a hydrogen bond and the addition of AM increased the strength of the hydrogen bond between the components of the film [22]. Furthermore, the absorption peak of N-H at 1414.59 cm^−1^ indicated the trace protein contained in the film, which proved that the protein/polysaccharide structure may exist in the film [23]. The absorption peak of films containing AM moved to the short wavenumber direction, which indicates that AM can enhance the stability of the protein/polysaccharide structure in the film.

In addition, FI-IR spectra showed that the positions and intensities of the absorption peaks changed among the films with different AM contents, but no new absorption peaks appeared. This means that the addition of AM likely causes no formation or the disappearance of chemical bonds, which causes a change of noncovalent bonds and some secondary bonds, and no chemical reaction occurs [24].

### 3.2. Microstructure of Bilayer Films

**Figure 2:**
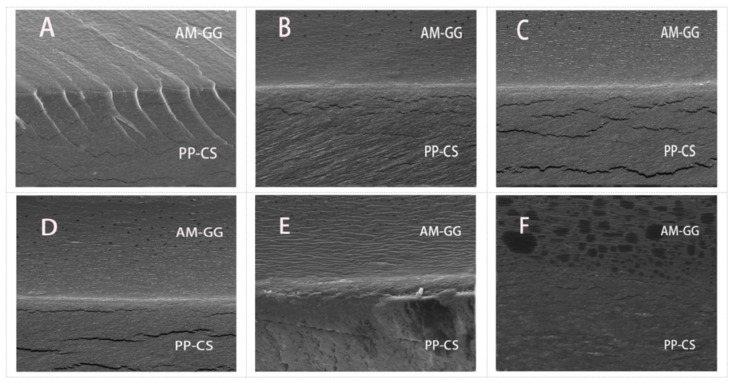
SEM cross-sectional images (2000×) of AM-GG/PP-CS bilayer films: 0.00% AM (**A**); 0.50% AM (**B**); 1.00% AM (**C**); 1.50% AM (**D**); 2.00% AM (**E**); 2.50% AM (**F**).

The microstructure of the film was observed by SEM (Figure 2). Results showed that the films with different AM concentrations all had obvious delamination boundaries, and the combination of structures was close without a noticeable fracture phenomenon. The films with different AM contents in the PP-CS layer displayed smooth cross-sections but had localized cracks. This indicates the addition of AM had no significant effect on the PP-CS layer [25].

Films without AM in the AM-GG layer showed obvious folds and rough cross-sections. However, as hydrophilic groups in AM formed hydrogen bonds with GG, the compatibility between AM and GG was increased, thus making the AM-GG layer more continuous, homogeneous and smooth [26]. The GG layer of the film after adding AM became more continuous, homogeneous and smooth, which was attributed to hydrophilic groups in AM forming hydrogen bonds with GG. Results showed that AM significantly improved the film microstructure, which is consistent with the research results of Woo et al. [6]. It is obvious from the figure that a large amount of AM small particles was dispersed in the AM-GG layer, which was gradually dense with the increase in AM content. Some small molecular particles were connected into pieces and were small particles after the AM content reached 2.50%. Dispersed particles in the microscale suggest the immiscibility of the polymer components, which possibly reduces mechanical and barrier properties [27]. 

### 3.3. Moisture Content (MC) and Thickness of Bilayer Films

**Figure 3:**
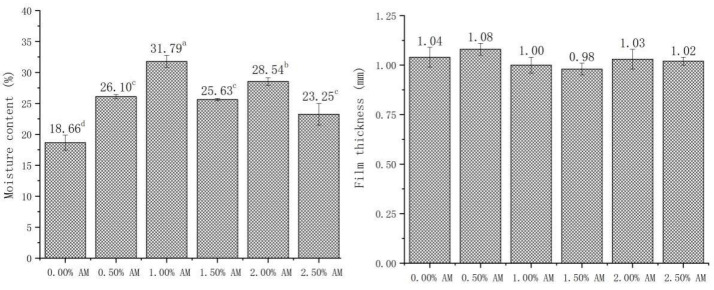
Moisture content and film thickness of AM-GG/PP-CS bilayer films. Data were submitted to one-way analysis of variance (ANOVA), different letters: Tukey’s post hoc method was applied to establish significant differences among samples (*p* < 0.05).

The MC has a significant influence on the properties of films, which can affect the mechanical properties of films through the plasticizing effect of water. Patrizia [10] found that the MC of films had a significant positive correlation with elasticity and ductility. As shown in Figure 3, the addition of AM significantly improved the MC of AM-GG/PP-CS bilayer films. The MC of AM-GG/PP-CS bilayer films with 1.00% and 2.00% AM is higher than other films, which increased by 31.79% and 28.54% compared with the film without AM. This is because the carbohydrate in AM has a large number of hydrophilic groups such as carboxyl, hydroxyl and aldehyde. These groups are easy to form hydrogen bonds with water molecules [28], thus enhancing the water-holding capacity of AM-GG/PP-CS bilayer films and significantly improving the MC [26]. In addition, according to the measurement results of membrane thickness, there was no significant difference in membrane thickness between the different groups of AM-GG/PP-CS bilayer films, indicating that the addition of AM had no significant effect on membrane thickness.

### 3.4. Mechanical Properties of Bilayer Films

**Figure 4:**
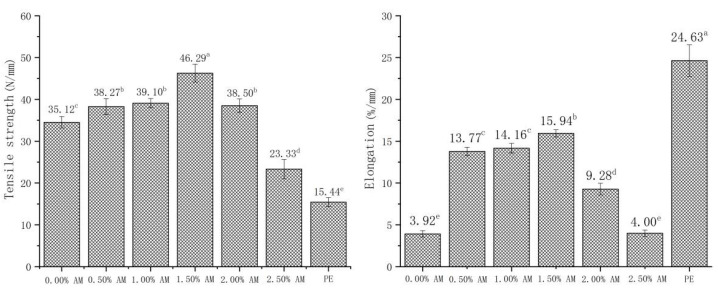
Tensile strength and elongation at break of AM-GG/PP-CS bilayer films. Data were submitted to one-way analysis of variance (ANOVA), different letters: Tukey’s post hoc method was applied to establish significant differences among samples (*p* < 0.05).

There were significant differences in tensile strength and elongation at break between AM-GG/PP-CS bilayer films with different AM concentrations (Figure 4). The tensile strength and elongation at break of films showed a trend of first increasing and then decreasing upon the increase in AM concentrations. This indicates that the addition of AM significantly improved the tensile strength of films. This is because the polyphenols in AM form hydrogen bonds and noncovalent hydrophobic interactions with GG [29,30]. The significant increase in elongation might be related to the increase in MC. Ismail [31] obtained similar results, showing that the moisture content of GG film was positively correlated with elongation, and the increase in MC had a significantly plasticizing effect on the film, thus strengthening the flexibility of the film. This was consistent with the results of the water content of AM-GG/PP-CS bilayer films mentioned above. However, the TS and EB of the film when the AM content exceeded 1.50% decreased significantly. This is because the small particles contained in the film gradually increased with the increase in AM content. Moreover, the network structure formed by GG could not cover all of the small particles contained. This resulted in the deterioration of the continuity of the AM-GG/PP-CS bilayer film structure and decrease in TS and EB.

**Figure 5:**
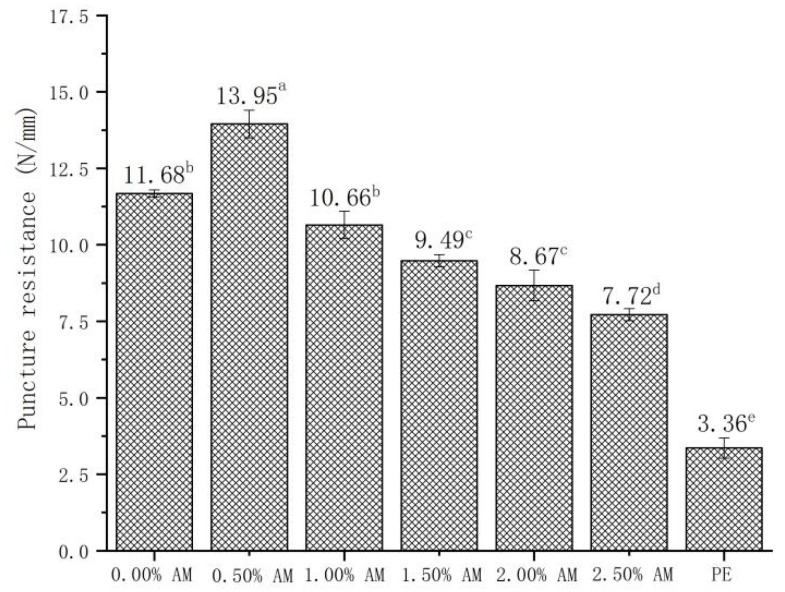
Puncture resistance of AM-GG/PP-CS bilayer films. Data were submitted to one-way analysis of variance (ANOVA), different letters: Tukey’s post hoc method was applied to establish significant differences among samples (*p* < 0.05).

The addition of AM had a significant effect on the puncture resistance of the film (Figure 5). Compared with the films without AM, the puncture resistance of AM-GG/PP-CS bilayer films with 0.50% AM increased by 19.41%. Moreover, the puncture resistance of films decreased with the increase in AM content. Results showed that the AM-GG/PP-CS bilayer films with low AM content had better puncture resistance. This mechanism was consistent with the effect of AM content on the TS of films [29,30]. Furthermore, the small particles contained in AM had a more significant impact on the puncture resistance of the film than on the TS. The puncture resistance after the AM content exceeded a 0.50% decrease [32]. In addition, compared with food-grade PE film, the puncture resistance of 0.50% AM film was significantly improved by 315.18%, which indicates that AM-GG/PP-CS bilayer films had better puncture resistance and were not easily damaged by the shape of the food.

The TS and EB of AM-GG/PP-CS bilayer films with the addition of 1.50% AM reached the maximum, which were 46.29 N/mm and 15.94%/mm, respectively. This indicates the film with 1.50% AM had the best mechanical strength and flexibility [33]. The maximum puncture resistance of AM-GG/PP-CS bilayer films was 13.95 N/mm when the content of AM was 0.50%, indicating that they had the optimum penetration resistance. Considering that AM-GG/PP-CS bilayer films were mainly applied in the field of packaging and preservation of fruits, vegetables and meat, the TS and EB of the film were more practical in terms of packaging and fresh-keeping effect than puncture resistance. Therefore, the mechanical experiment results showed that the mechanical properties of AM-GG/PP-CS bilayer films at the amount of 1.50% AM were the best. 

### 3.5. Water Vapor Permeability (WVP) and Oil Permeability (OP)

**Figure 6:**
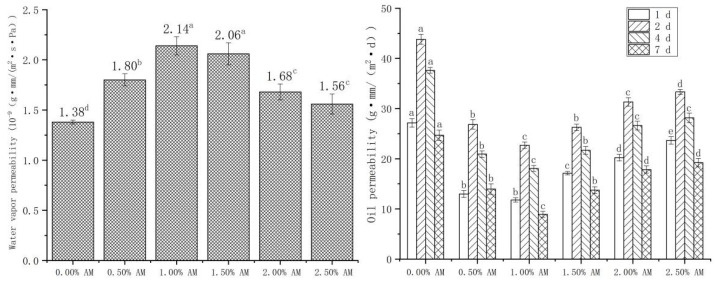
Water vapor permeability and oil permeability of AM-GG/PP-CS bilayer films. Data were submitted to one-way analysis of variance (ANOVA), different letters: Tukey’s post hoc method was applied to establish significant differences among samples (*p* < 0.05).

WVP and OP show the water and oil resistance properties of the film, respectively. Moisture and oil content significantly affect the quality and shelf life of food; thus, good water and oil resistance are important indicators to evaluate the properties of films [34]. The addition of AM had a significant impact on the WVP and OP of the film (Figure 6). The WVP of the film with the increase in AM content increased firstly and then decreased. The WVP of the film reached a maximum value of 2.14 × 10^−9^ g·mm/(m^2^·s·Pa) at 1.00% AM content, then significantly increased by 55.07% compared with the film without AM. Hydrophilic groups, such as hydroxyl and carboxyl contained in AM, enhanced the polar interaction between molecular properties of the film and improved the free volume [35,36]. Large numbers of hydroxyl groups readily absorb water from the environment, improving plasticization and permeability [37]. The increased space allows hydrogen to pass through the film more easily, thus improving the hydrophilicity of the film. Similarly, the addition of glycerol to pullulan/GG composite film significantly improved the hydrophilicity and WVP of the film [38].

In addition, the effect of AM on the OP of films was opposite to that of WVP. With the increase in AM concentrations, OP showed a significant trend of first decreasing and then increasing. When the content of AM was 1.00%, the OP reached the minimum value of 22.71 g·mm/(m^2^·d) at 2 d, which was 48.16% lower than that of the film without AM. The reason was similar to the effect of AM on the water resistance of the film [35]. The addition of AM led to the enhancement of the polar interaction between the molecular properties of the film, which inhibited the passage of nonpolar edible oil and reduced the OP of the film, thus enhancing the oil resistance of the film. The OP of the same sample showed a trend of increasing first and then decreasing with the extension of time, and the maximum OP appeared at 2 d. This demonstrates that the OP of the film was the largest in the first 2 d, then gradually decreased with the extension of time.

### 3.6. Color Changes of AM-GG/PP-CS Bilayer Films at Different pH Values

**Figure 7:**
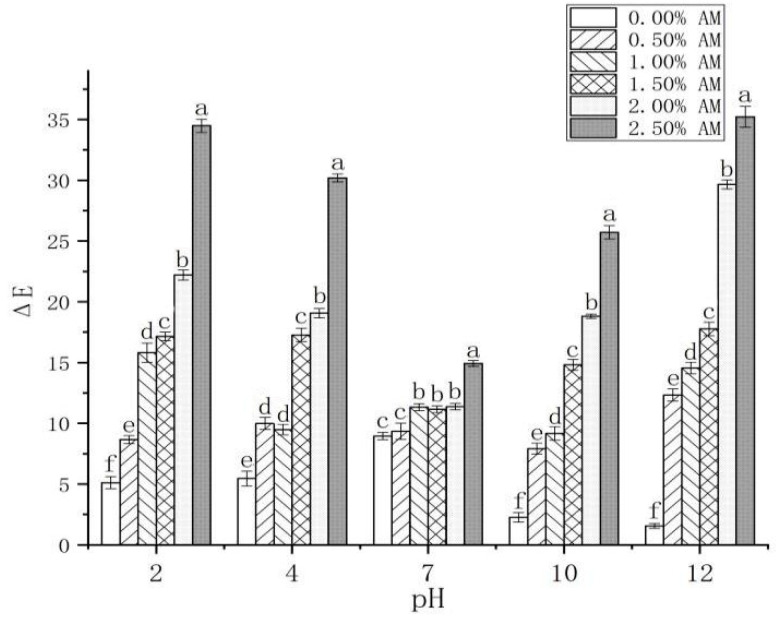
Changes of AM-GG, PP-CS bilayer edible films at different pH values. Data were submitted to one-way analysis of variance (ANOVA), different letters: Tukey’s post hoc method was applied to establish significant differences among samples (*p* < 0.05).

The color changes of the AM-GG/PP-CS bilayer edible composite film under different pH values (2, 4, 7, 10 and 12) were investigated (Table 1). Compared with the composite film without AM, the composite film with AM was red and green under acidic (pH 2, pH 4) and alkaline (pH 10, pH 12) conditions, respectively. Furthermore, with the gradual increase in the pH value from 2 to 12, the color of the AM-GG/PP-CS bilayer edible composite film with different AM contents changed from red to green. This is attributed to the fact that AM contains a large number of anthocyanins, anthocyanins and other substances [39], which could be used as chromogenic substances to make the membrane show different colors under different pH conditions [1]. The color of the composite film with different AM contents under the same pH condition also showed a significant difference (Table 1; Figure 7). Compared with the other experimental groups, the color of the 2.50% AM film was deeply affected by pH, and the discoloration degree was the highest, indicating that it possessed the optimum pH sensitivity to discoloration. The color of AM-GG/PP-CS films was highly sensitive to different pH values, which exhibited different colors with the change of pH value. Results showed that the films had the potential to be used as indicators of food freshness, which was attributed to the fact that they can reflect the change of food pH value and judge the freshness of food through the color change of the film.

### 3.7. Fresh-Keeping Effect of AM-GG/PP-CS Bilayer Edible Composite Film on Fresh Pork

**Figure 8:**
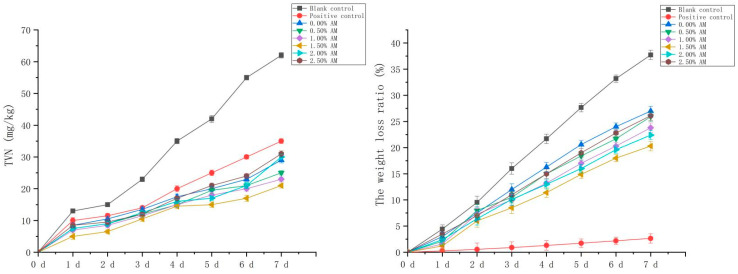
TVN basic nitrogen in pork under different fresh-keeping methods and ratio of weight loss in pork.

The fresh-keeping performance of pork with the AM-GG/PP-CS bilayer edible composite film with different AM contents was evaluated. The pork without fresh-keeping treatment was used as the blank control, and the pork fresh-keeping with food fresh-keeping film was used as the positive control. TVN is a kind of salt-based nitrogen produced by the combination of nitrogen-containing alkaline substances and organic acids after the protein in meat is decomposed by microorganisms. Its content is closely related to the growth and reproduction of microorganisms in meat, which can reflect the changes of freshness and quality during meat storage [1]. Therefore, the content of TVN in pork was an important evaluation index to reflect the degree of pork corruption. Compared with the blank control, the AM-GG/PP-CS films with different AM contents significantly inhibited the production of the TVN of pork during storage (Figure 8), indicating that the composite film had excellent fresh-keeping ability and significantly inhibited the corruption and deterioration of fresh pork. The TVN content of fresh pork with the increase in AM content increased initially and then decreased. Compared with the blank control, the composite film with 1.50% AM content had the optimum inhibitory effect on TVN, and the TVN content was significantly reduced by 66.13% on the 7th day of storage. This was due to the existence of AM in the composite film, which effectively inhibited the growth and reproduction of spoilage bacteria and played a fresh-keeping role in fresh pork. Furthermore, compared with the positive control, the inhibition effect of the TVN of the composite film with different AM contents performed more significantly. The TVN content of the composite film decreased significantly by 40.00% compared with the positive control on the 7th day of storage. Results showed that the fresh-keeping ability of the composite film was significantly stronger than that of the food film.

The weight loss and appearance changes of fresh pork under different storage conditions were analyzed (Figure 8; Table 2). Results showed that the weight loss of fresh pork without fresh-keeping treatment during the storage time of 7 days reached 37.72%. The excessive weight loss reduced the moisture content of pork, making the meat hard and dry, which significantly reduced the sensory quality of fresh pork. Compared with the blank group, the weight loss of fresh pork decreased significantly after the fresh-keeping treatment of fresh pork with the composite film. That was consistent with the research results of the water vapor transmission coefficient of composite film, indicating the composite film improved the weight loss of fresh pork during storage and improve the quality. However, compared with the positive control, the weight loss ratio of fresh pork preserved with the composite film increased significantly. This suggests the composite film was slightly inferior to the food preservation film in reducing the weight loss of fresh pork.

## 4. Conclusions

The quality of the pH-sensitive bilayer hydrogel film containing AM was significantly improved. Results showed that GG in the film forms a strong hydrogen bond interaction with the hydroxyl group, carboxyl group and others in AM. The film microstructure containing AM was smoother and denser, which possessed the optimum characteristic of MC, TS, puncture resistance and oil resistance. However, the water resistance of the film decreased with the enhancement of the polarity of the film. In addition, the active substances (such as anthocyanins and polyphenols) in AM gave the film excellent functional characteristics, which made it possess the ability of pH-sensitive discoloration and reduce the content of TVN in pork.

In conclusion, AM played an important role in the construction of AM-GG/PP-CS bilayer films. Compared with other films, the film with 1.5%AM content had the optimum mechanical properties and good visual pH-sensitive discoloration. It was more suitable for food preservation packaging comprehensive application of performance analysis and can be used as an indicator of food freshness to detect the quality changes of food in the storage process.

## Figures and Tables

**Table 1:** Color changes of AM-GG, PP-CS bilayer edible films at different pH values.

Samples	pH					
	Blank Control	2	4	7	10	12
0.00% AM	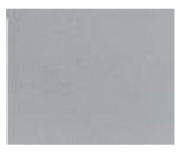	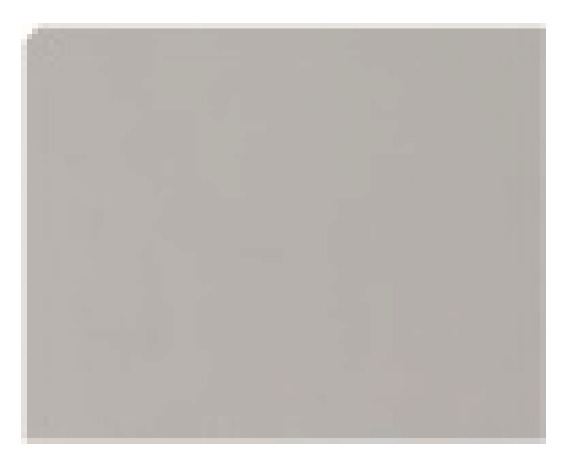	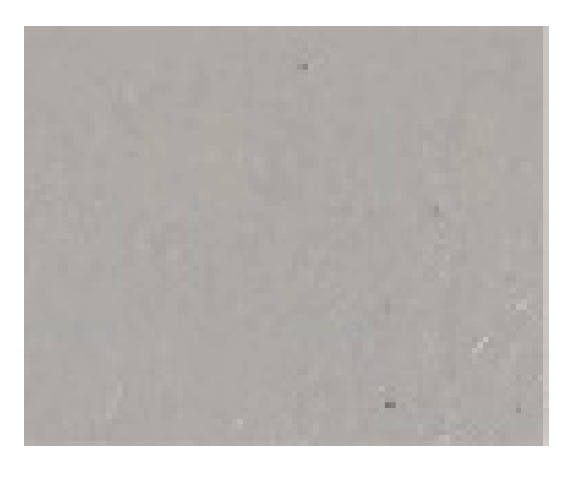	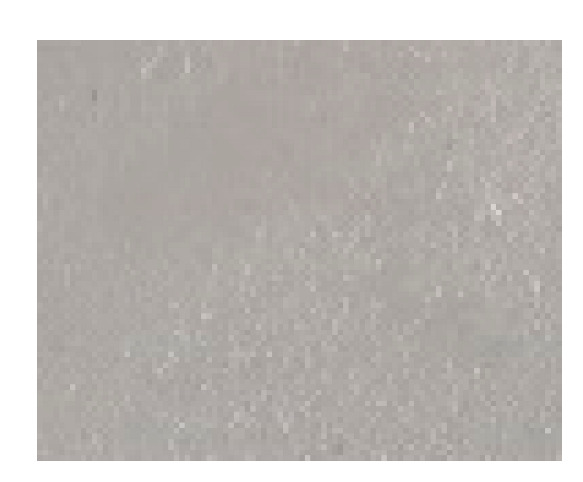	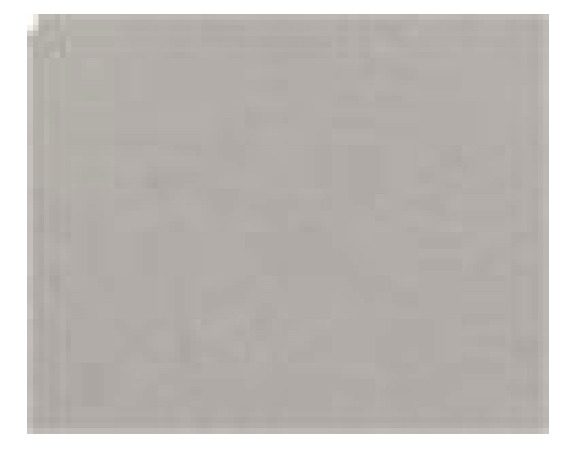	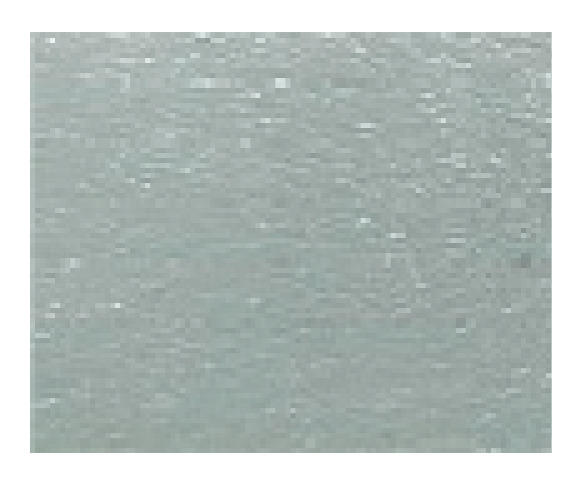
0.50% AM	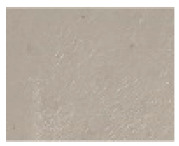	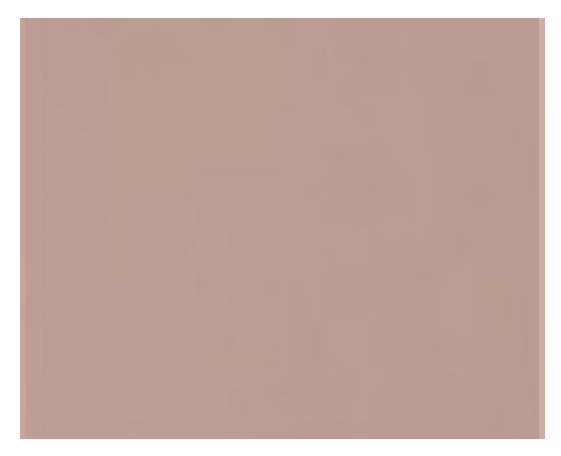	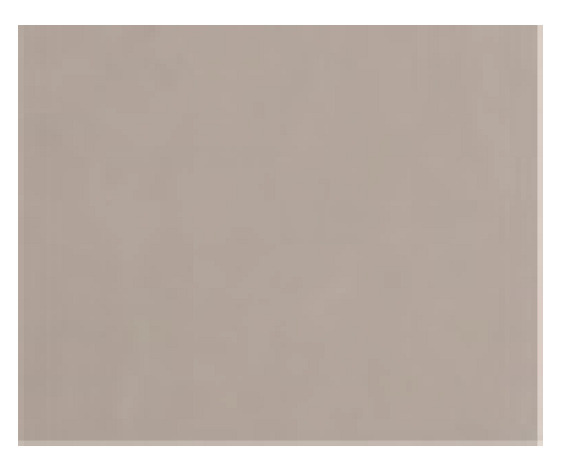	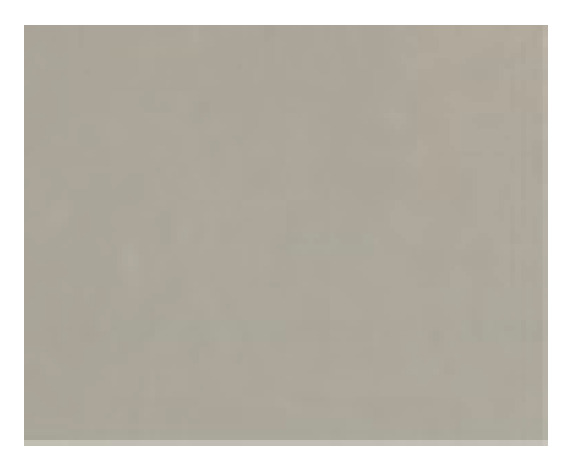	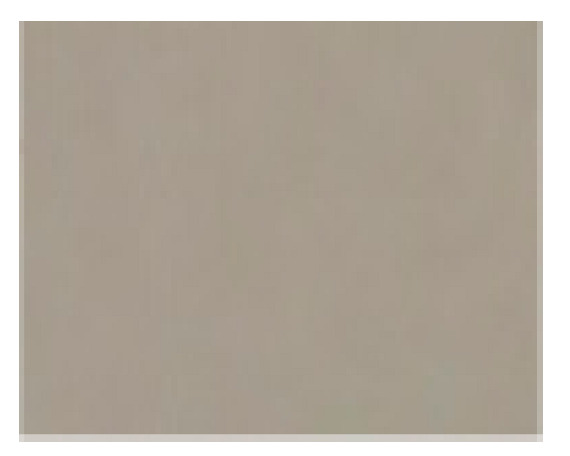	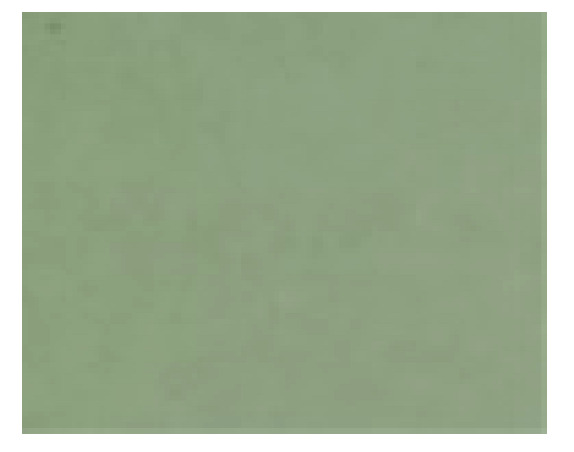
1.00% AM	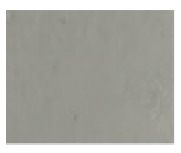	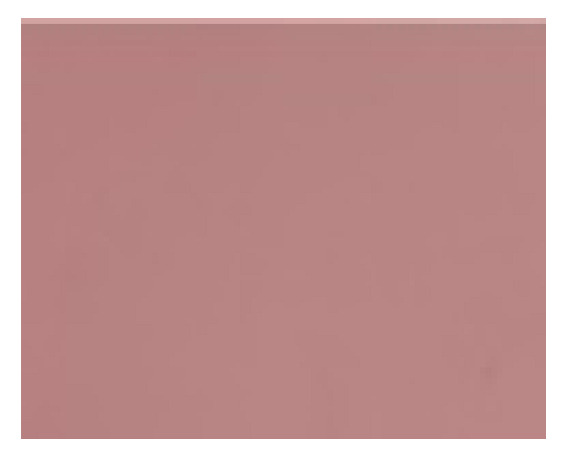	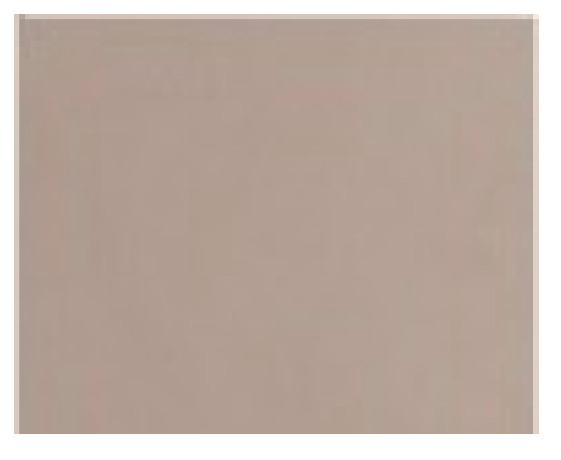	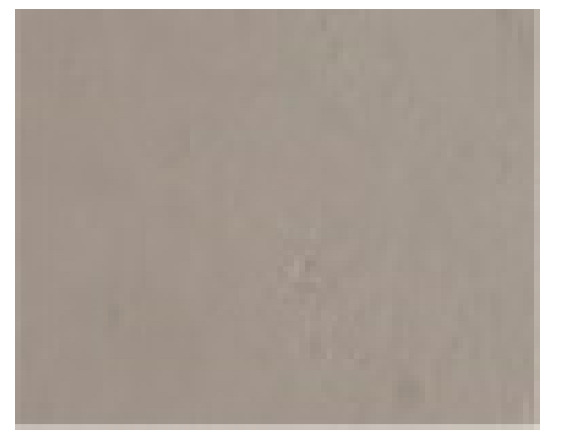	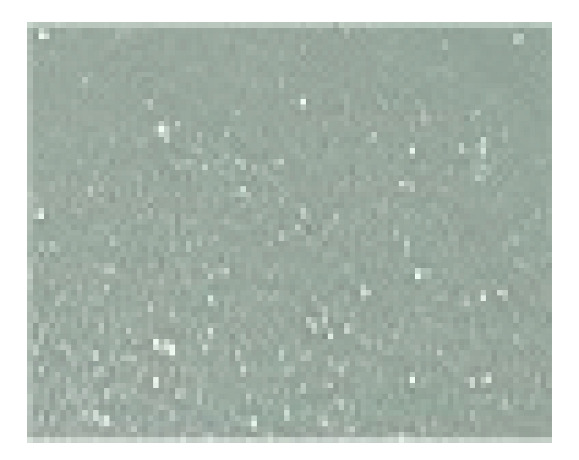	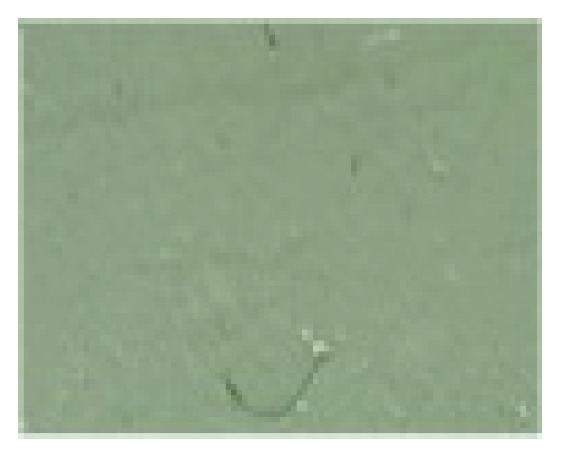
1.50% AM	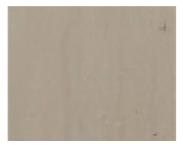	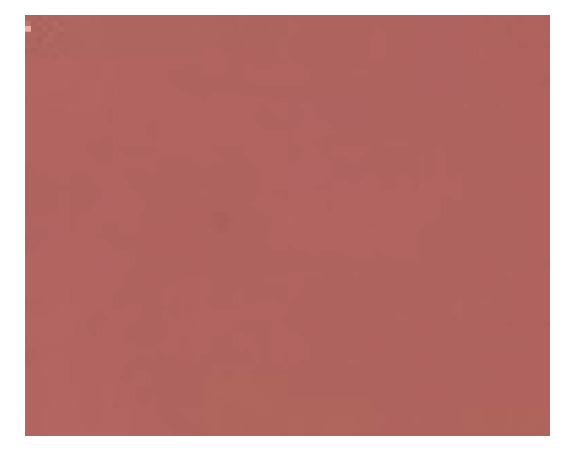	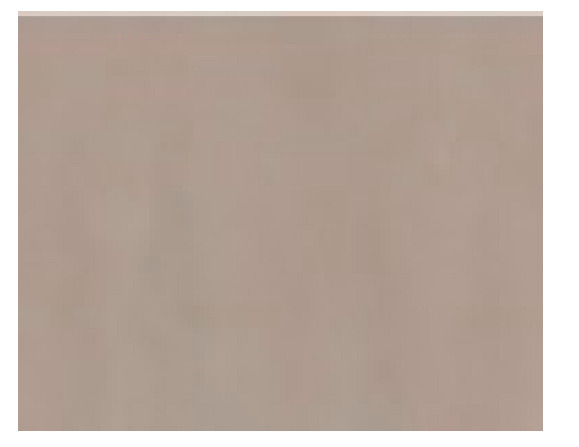	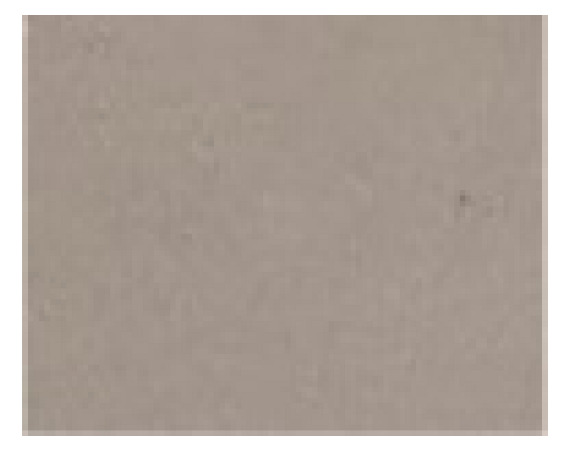	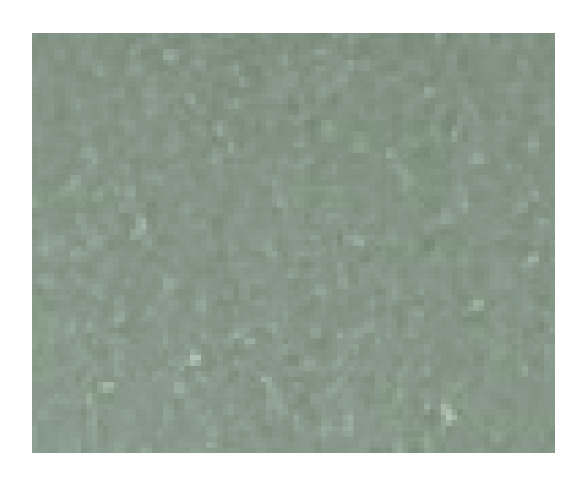	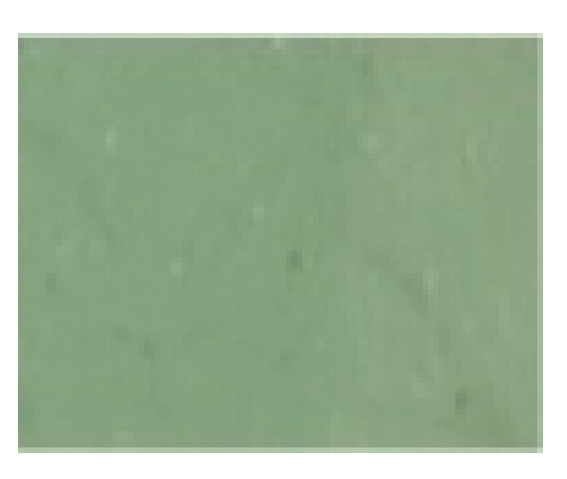
2.00% AM	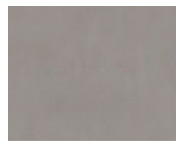	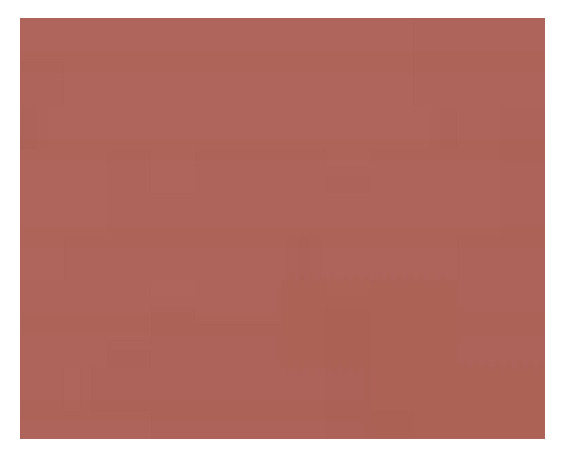	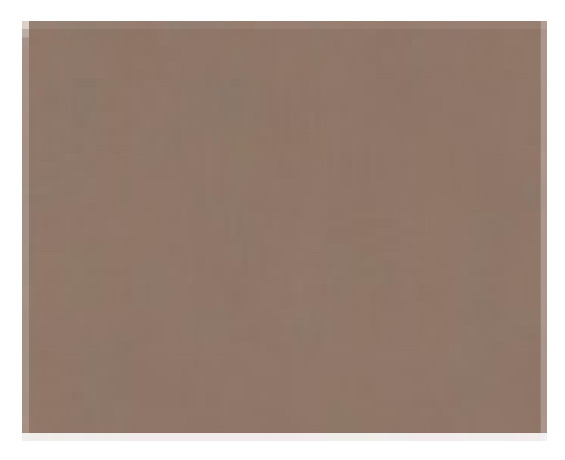	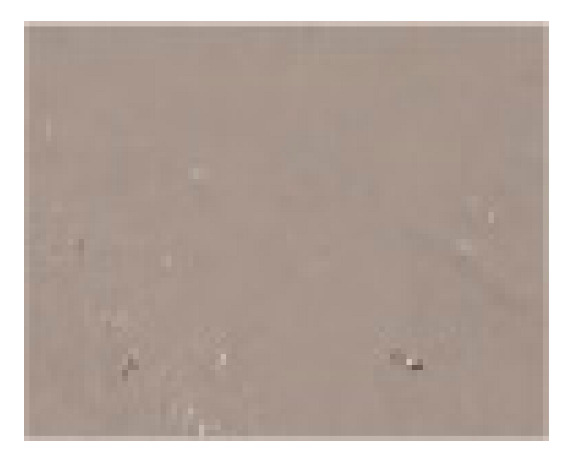	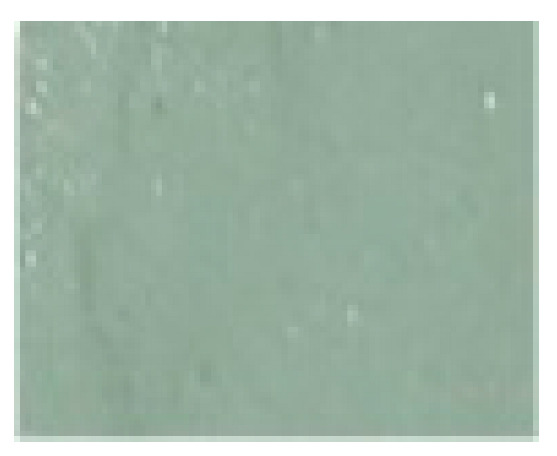	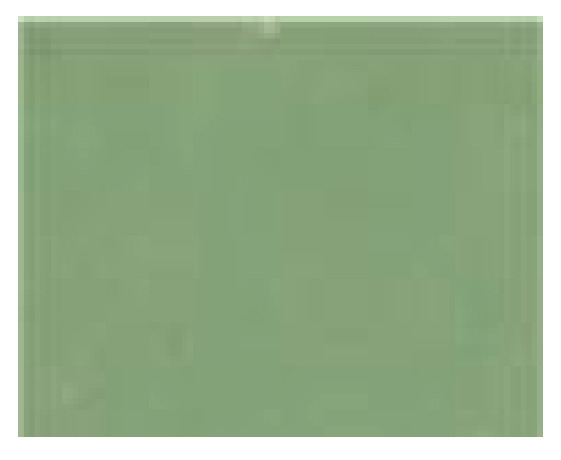
2.50% AM	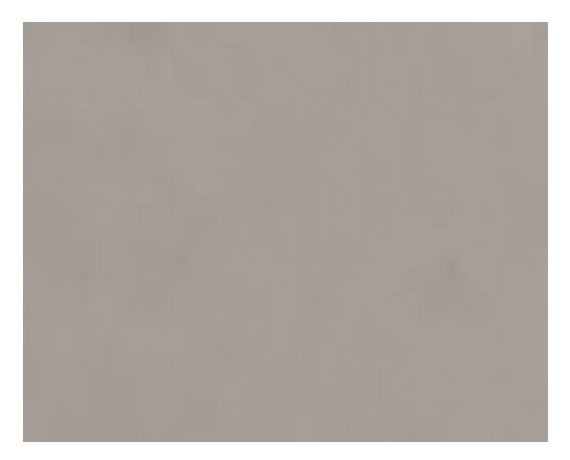	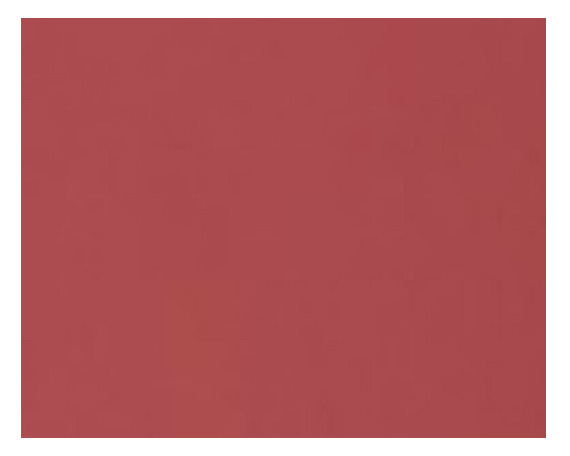	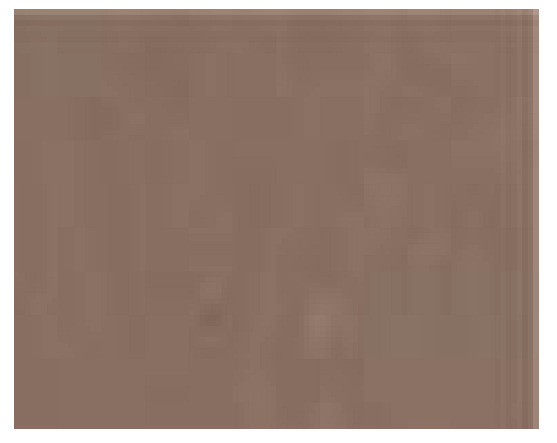	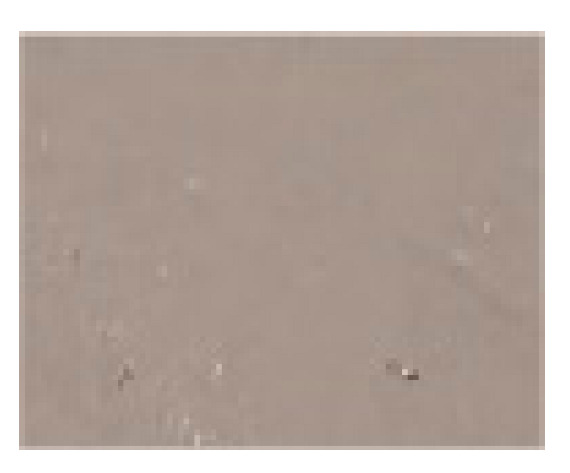	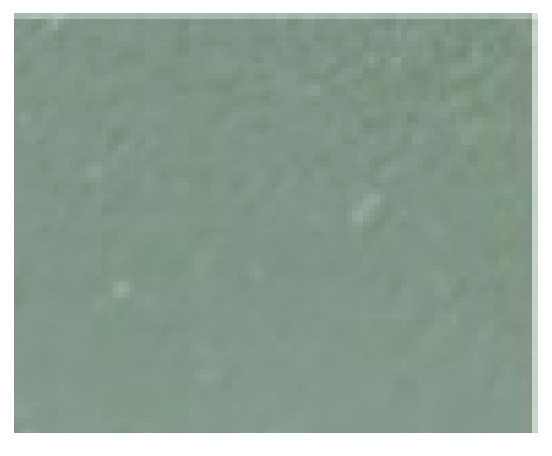	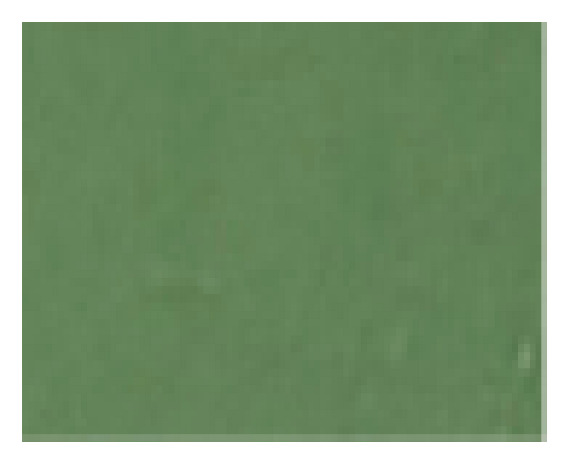

**Table 2:** Changes of appearance of pork in pork under different fresh-keeping methods.

	Samples	Blank Control	Positive Control	0.00% AM	0.50% AM	1.00% AM	1.50% AM	2.00% AM	2.50% AM
Days									
0 d		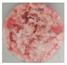	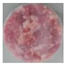	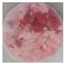	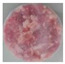	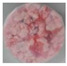	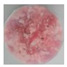	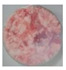	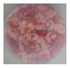
1 d		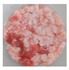	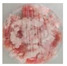	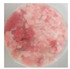	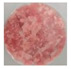	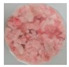	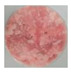	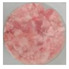	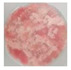
2 d		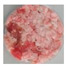	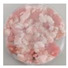	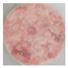	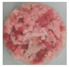	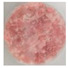	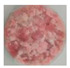	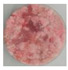	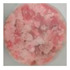
3 d		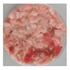	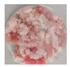	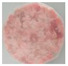	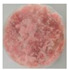	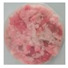	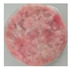	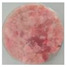	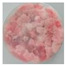
4 d		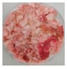	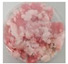	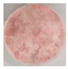	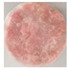	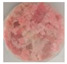	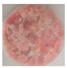	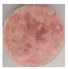	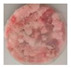
5 d		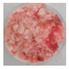	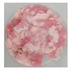	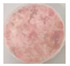	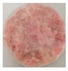	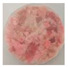	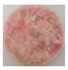	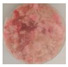	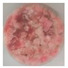
6 d		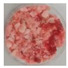	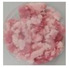	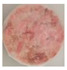	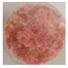	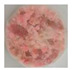	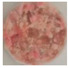	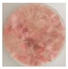	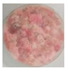
7 d		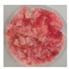	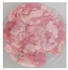	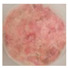	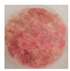	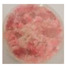	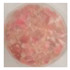	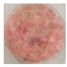	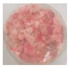

## Data Availability

Data are contained within the article.
